# Study on the association between the low-density lipoprotein to high-density lipoprotein ratio and clinicopathological characteristics of breast cancer: A retrospective analysis incorporating subgroup evaluations by age, body mass index, and menopausal status

**DOI:** 10.1371/journal.pone.0361764

**Published:** 2026-10-29

**Authors:** Lei Dong, Can Liu, Lixin Li, Xiaotong Zhang, Wei Xu, Gaosai Dai

**Affiliations:** 1 Department of Breast Surgery, Qilu Hospital of Shandong University, Jinan, Shandong, China; 2 Department of Thyroid and Breast Surgery, Jinan Third People’s Hospital, Jinan, Shandong, China; 3 Department of Breast Surgery, Jinan Maternity Child Careand Hospital, Jinan, Shandong, China; 4 Minimally invasive surgery, First People’s Hospital of Jinan, Jinan, Shandong, China; 5 Department of Plastic Surgery, Qilu Hospital (Qingdao), Cheeloo College of Medicine, Shandong University, Qingdao, Shandong, China; University of California Riverside, UNITED STATES OF AMERICA

## Abstract

Breast cancer (BC) represents the most prevalent malignant tumor among women globally, with its pronounced heterogeneity presenting significant challenges for precision diagnosis and treatment. Aberrant lipid metabolism, particularly the ratio of low-density lipoprotein to high-density lipoprotein (LHR), has garnered considerable interest in relation to BC development and progression. Nevertheless, the association between LHR and clinicopathological characteristics remains inadequately explored. To address this gap, the present retrospective study analyzed 583 BC patients who underwent surgical treatment at Qilu Hospital of Shandong University. Preoperative blood lipid profiles and clinicopathological data were systematically collected to investigate the association between LHR and critical features, including molecular subtypes. The findings indicated that LHR levels were significantly elevated in patients aged 46 years or older, postmenopausal women, and individuals with a body mass index of 25 kg/m^2^ or higher. Subgroup analyses incorporating age, menopausal status, and BMI suggested potential interactions between these clinical factors and LHR. Although univariate analysis demonstrated an association between LHR and certain clinicopathological features, multivariate logistic regression analysis revealed that menopausal status, rather than LHR, was the sole independent predictor of ER-positive and PR-positive status. Therefore, LHR should not be considered an independent predictive biomarker for receptor status. In conclusion, although the LHR demonstrates statistically significant associations with certain clinicopathological characteristics, its modest effect sizes and discriminative ability close to chance indicate limited clinical utility as an independent biomarker. These preliminary findings highlight the need for future research focused on identifying reliable metabolic signatures. Such studies should consider utilizing multi-marker panels instead of relying exclusively on individual lipid metrics for risk stratification in breast cancer.

## Introduction

Breast cancer (BC) has long been recognized as the most prevalent malignant tumor among women worldwide, representing a significant threat to women’s health and survival [1–3]. Over the past two decades, the annual incidence of BC has increased by approximately 0.33%, with the global number of cases rising from 876,990 to 2,002,350 [4]. BC is a highly heterogeneous disease that can be classified into multiple subtypes based on the immunohistochemical expression of hormone receptors, including estrogen receptor-positive (ER+), progesterone receptor-positive (PR+), human epidermal growth factor receptor 2-positive (HER2+), and triple-negative BC (TNBC), which lacks all three receptors [5–7]. These subtypes demonstrate substantial differences in gene expression profiles, which influence tumor aggressiveness, treatment response, and prognosis [8–11]. The heterogeneity of BC poses considerable challenges to advancing the understanding of its pathological characteristics and underlying mechanisms.

Abnormal blood lipid levels are implicated in the pathogenesis of BC and have garnered significant attention due to their ease of measurement and potential for therapeutic intervention [12–14]. Cholesterol metabolism encompasses the regulation of low-density lipoprotein (LDL), high-density lipoprotein (HDL), and total cholesterol (TC). LDL, a major constituent of TC, has been demonstrated to facilitate cancer cell proliferation and migration by modulating the tumor microenvironment and hormone signaling pathways [15–18]. Conversely, HDL is generally regarded as protective against BC owing to its anti-inflammatory and antioxidant properties [19–21]. Nonetheless, the association between lipid parameters and BC risk remains complex and not fully elucidated. Some studies report a positive association between elevated LDL levels and BC risk, potentially mediated through oxidative modification, activation of pro-inflammatory signaling pathways, and provision of cholesterol essential for tumor growth; however, other investigations have failed to identify a significant relationship [22,23]. Similarly, while higher HDL levels are typically linked to a decreased risk of BC, reduced HDL concentrations may compromise its anti-inflammatory, antioxidant, and reverse cholesterol transport functions, thereby exacerbating the deleterious effects of LDL-C and increasing BC risk [24]. Despite these observations, the evidence remains inconsistent. In summary, the precise roles of lipid metabolism markers such as TC, HDL, and LDL in BC development and progression remain contentious, and conflicting findings hinder the establishment of definitive conclusions.

A comprehensive understanding and effective management of BC risk necessitate an emphasis on lipid metabolism, particularly the dynamic equilibrium between LDL and HDL. Recent research suggests that the LDL to HDL ratio (LHR) may serve as a significant prognostic indicator for BC, demonstrating greater predictive accuracy than the assessment of LDL or HDL levels independently in certain populations [25–28]. Despite multiple investigations highlighting the biological relevance of LHR in tumor metabolism, there remains a paucity of systematic research utilizing real-world data to directly assess the relationship between LHR and the clinicopathological characteristics of BC.

Given the steadily rising incidence of BC among Chinese women, systematically investigating the relationship between the LHR and the clinicopathological characteristics of BC is of considerable theoretical significance and practical relevance. This research contributes to the enhancement of risk assessment models and facilitates the advancement of more precise prevention and treatment strategies.

## Methods

### Patients

This study was conducted in accordance with the principles outlined in the Declaration of Helsinki and received approval from the Ethics Committee of Shandong University School of Medicine (Protocol No.: KYLL-202212-021). Given the retrospective design of the study, which utilized solely anonymized data extracted from medical records and involved no direct patient interaction or interventions, the ethics committee waived the requirement for written informed consent. The data were accessed for research purposes on 03/01/2024. All data were fully anonymized prior to access, authors don’t have access to information that could identify individual participants during or after data collection.

The inclusion criteria were as follows:

(1) Patients diagnosed with unilateral BC who underwent initial surgical treatment at Qilu Hospital of Shandong University between January 1, 2020, and January 31, 2024; (2) BC diagnosis confirmed by routine pathological examination; (3) no receipt of neoadjuvant therapy before surgery; (4) availability of a complete blood lipid profile within one week prior to surgery; (5) no prior use of lipid-lowering agents or other medications known to affect blood lipid levels; and (6) absence of comorbid conditions potentially influencing blood lipid levels, including hypertension, coronary heart disease, diabetes mellitus, thyroid disorders, cerebral infarction, chronic viral hepatitis, and other relevant diseases.

The exclusion criteria were as follows:

(1) Male BC patients; (2) patients with recurrent BC; (3) patients with bilateral BC or a history of contralateral BC; (4) BC patients who were pregnant or breastfeeding; (5) patients diagnosed with inflammatory BC or breast sarcoma; (6) patients with incomplete or missing critical clinical data; (7) patients who received neoadjuvant therapy prior to surgery; (8) patients who had used lipid-lowering agents or other medications influencing lipid levels; and (9) patients with comorbid conditions potentially affecting lipid metabolism, including hypertension, coronary heart disease, diabetes mellitus, thyroid disorders, cerebral infarction, chronic viral hepatitis, among others. Data on general clinical characteristics, preoperative lipid profiles, and routine pathological findings were obtained through the medical record inquiry system, laboratory results inquiry system, and pathology inquiry system at Qilu Hospital of Shandong University.

The clinical data encompassed the following variables: age (years), body mass index (BMI, kg/m^2^), family history of BC, menopausal status, pathological type, tumor size (cm), presence or absence of lymph node metastasis, estrogen ER status, PR status, HER2 status, Ki-67 expression level, and blood lipid indicators. Tumor size is defined as the greatest diameter measured from the postoperative pathological specimen. For the analysis of categorical variables, tumor size is classified into two groups: ≤ 2 cm (Stage T1) and > 2 cm (Stage T2 and above). Tumor histological grading was classified according to the Scarff-Bloom-Richardson (SBR) system for grade I, II, and III. Postoperative paraffin-embedded pathological analysis was used to determine lymph node status; cases with lymph node metastasis were designated as lymph node-positive, whereas those without metastasis were designated as lymph node-negative. ER and PR status were assessed via immunohistochemical staining, with positivity defined as nuclear staining in ≥1% of tumor cells and negativity as nuclear staining in <1% of tumor cells. HER2 status was determined as follows: patients exhibiting HER2+++ staining were classified as HER2 positive, while those with HER2+ or 0 staining were classified as negative. For cases with HER2++ staining, in situ hybridization results were incorporated; if HER2 gene amplification was detected, the case was classified as positive, otherwise as negative. Ki-67 expression was categorized as low (≤ 14%) or high (> 14%). All postoperative pathological data were obtained from the pathology department of our hospital, thereby ensuring data reliability and consistency. Each pathological specimen underwent thorough analysis and evaluation, with every pathology report reviewed and diagnosed by a minimum of two senior pathologists to guarantee the accuracy and professionalism of the findings.

### Determination of lipidogram in patients

All enrolled subjects underwent peripheral venous blood collection in the morning following a 12-hour fasting period prior to surgery. Blood lipid levels were measured after this fasting interval. All blood samples were processed consistently by the Clinical Laboratory Department of Qilu Hospital to ensure uniformity in test results. The patients’ blood lipid levels were analyzed using the Cobas 8000 (701) system (Roche Diagnostics, Germany).

### Statistical analysis

We performed descriptive analyses on the clinical data of all patients included in the study. For continuous variables exhibiting a normal distribution, comparisons were conducted using t-tests, with results presented as mean ± standard deviation (SD). For continuous variables not conforming to a normal distribution, Mann-Whitney U tests were employed, and data were reported as median and interquartile range (IQR). These analyses aimed to assess differences in LHR levels among patients stratified by menopausal status (menopausal versus non-menopausal), age groups, body mass index (BMI), and molecular pathological expression status, including ER, PR, HER2, and Ki67 levels.

Univariate logistic regression analysis was performed, and variables with a significance level of p < 0.05 were subsequently included in a multivariate logistic regression analysis to identify independent factors influencing binary outcomes. Odds ratios (ORs) and their corresponding 95% confidence interval (CI) were employed to assess the effects of potential risk factors on the outcomes. A p-value of less than 0.05 was considered indicative of statistical significance.

Subsequently, receiver operating characteristic (ROC) curves were constructed, and the area under the curve (AUC) with a 95% CI was employed to evaluate the predictive performance of age, BMI, menopausal status and LHR for ER, PR, and HER2 status. All data analyses were conducted using Origin software, and all figures were produced with GraphPad Prism 9.

## Results

### Characteristics of patients

Based on the specified inclusion and exclusion criteria, this study enrolled a total of 583 BC patients. The patients’ ages ranged from 24 to 87 years, with a median age of 46 years. Among these patients, 356 (61.06%) were premenopausal at diagnosis, while 227 (38.94%) were postmenopausal. The predominant pathological subtype was invasive ductal carcinoma, representing 81.48% (475/583) of cases. The largest proportion of patients were diagnosed at pathological stage II, accounting for 55.57% (324/583). Lymph node metastasis was observed in 38.76% (226/583) of the cohort. Molecular pathological analysis revealed that 436 patients (74.79%) were ER+, 402 (68.95%) were PR+, and 167 (28.64%) were HER2+. Furthermore, 69.81% (407/583) of patients exhibited Ki67 expression levels exceeding 14%. Detailed clinical characteristics of the enrolled patients are presented in Table 1.

**Table 1:** Characteristics of breast cancer patients.

Clinical Features	Group	Quantity and Proportion
Age	Median age	46
	Range	24-87
BMI	<18.5	6 (1.03%)
	18.5-23.9	228 (39.11%)
	24-28	232 (39.79%)
	>28	117 (20.07%)
Family history of BC	Yes	5 (0.86%)
	No	578 (99.14%)
Menopausal status	Premenopausal	356 (61.06%)
	Postmenopausal	227 (38.94%)
Pathological type	Invasive ductal carcinoma	475 (81.48%)
	Invasive lobular carcinoma	35 (6.00%)
	Intraductal carcinoma	73 (12.52%)
Pathological stage	I	78 (13.38%)
	II	324 (55.57%)
	III	181 (31.05%)
Tumor size	≤2 cm	331 (56.78%)
	>2 cm	252 (43.22%)
Lymph node metastasis	Yes	226 (38.77%)
	No	357 (61.23%)
ER	Positive	436 (74.79%)
	Negative	147 (25.21%)
PR	Positive	402 (68.95%)
	Negative	181 (31.05%)
HER2	Positive	167 (28.64%)
	Negative	416 (71.36%)
Ki67	≤14%	176 (30.19%)
	>14%	407 (69.81%)

### LHR values of different characteristics

Based on calculations derived from the aforementioned formulas, the study findings concerning the distribution of various population characteristics indicated that patients younger than 46 years exhibited an LHR value of 1.83. Conversely, patients aged 46 years and older generally demonstrated elevated lipid levels, with an LHR value of 2.17 (P < 0.001), the median difference is 0.24 (95% CI: 0.14–0.34). Postmenopausal patients presented significantly higher LHR levels compared to premenopausal patients, with values of 2.26 and 2.02, respectively (P < 0.001), the median difference is −0.24 (95% CI: −0.29–-0.19). Furthermore, patients with a body mass index (BMI) ≥ 25 kg/m^2^ had an LHR level of 2.33, which was significantly greater than the 2.00 observed in patients with a BMI < 25 kg/m^2^ (P < 0.001), the median difference is 0.33 (95% CI: 0.22–0.44). These results are summarized in Table 2.

**Table 2:** LHR values of different characteristics.

Clinical Features	Group	LHR values	P value
Age	≥46	2.17 (1.71, 2.27)	<0.001
	<46	1.83 (1.50, 2.26)	
Menopausal status	Premenopausal	2.02 (1.58, 2.48)	<0.001
	Postmenopausal	2.26 (1.80, 2.75)	
BMI	≥25 kg/m^2^	2.33 (1.90, 2.83)	<0.001
	<25 kg/m^2^	2.00 (1.57, 2.47)	

### LHR values of different pathological characters of BC

Patients presenting with tumor size exceeding 2 cm exhibited a LHR of 2.20, which was significantly higher than the LHR of 2.10 observed in patients with tumor size of 2 cm or less (P < 0.001), the median difference is −0.10. (95% CI: −0.17–-0.03). No significant differences in LHR levels were identified among histological grades I, II, and III. Additionally, there was no statistically significant difference in LHR between patients with lymph node metastasis and those without, as detailed in Table 3.

**Table 3:** LHR values of different pathological characters of BC.

Clinical Features	Group	LHR values	P value
Tumor size	≤2 cm	2.10 (1.64,2.61)	<0.001
	>2 cm	2.20 (1.75,2.69)	
Histological grades	I	2.18 (1.79,2.59)	0.820
	II	2.14 (1.66,2.62)	
	III	2.14 (1.71,2.69)	
Lymph node metastasis	Yes	2.15 (1.66,2.70)	0.438
	No	2.14 (1.69,2.62)	

### LHR values of different pathological types of BC

In patients positive for ER, the LHR level was 2.13, which was significantly different from the level of 2.18 observed in ER-negative patients (P = 0.047), the median difference is −0.05. (95% CI: −0.13–0.03). Similarly, consistent with the ER subtype results, the LHR level in PR-positive patients was 2.13, demonstrating a significant difference compared to 2.18 in PR-negative patients (P = 0.024), the median difference is −0.05. (95% CI: −0.09–-0.01). No statistically significant differences in LHR levels were observed between HER2-positive and HER2-negative patients (P > 0.05). Additionally, variations in Ki67 expression levels were not associated with significant differences in LHR levels (P > 0.05) (Table 4).

**Table 4:** LHR values of different pathological types of BC.

Clinical Features	Group	LHR values	P value
ER	Positive	2.13 (1.67, 2.62)	0.047
	Negative	2.18 (1.72, 2.74)	
PR	Positive	2.13 (1.66, 2.62)	0.024
	Negative	2.18 (1.72, 2.70)	
HER2	Positive	2.15 (1.68, 2.65)	0.790
	Negative	2.14 (1.68, 2.64)	
Ki67	Positive	2.15 (1.70, 2.62)	0.432
	Negative	2.14 (1.67, 2.66)	

### Subgroup analysis

Given the potential influence of age, menopausal status, and BMI on lipid levels, this study conducted additional subgroup analyses based on these variables. Initially, statistical analyses were performed on subgroups stratified by age. The results indicated that among patients younger than 46 years, the LHR was significantly higher in PR-negative patients compared to PR-positive patients (P = 0.016 < 0.05). No significant differences in lipid-derived indicators were observed across other pathological subtypes (Table 5).

**Table 5:** Analysis results of LHR values for age subgroups.

Clinical Features	Group	LHR values	P value
Age ≥ 46	ER-positive	2.17 (1.71, 2.67)	0.155
	ER-negative	2.20 (1.72, 2.76)	
	PR-positive	2.16 (1.70, 2.65)	0.158
	PR-negative	2.19 (1.72, 2.74)	
	HER2-positive	2.17 (1.70, 2.68)	0.924
	HER2-negative	2.17 (1.71, 2.67)	
	Ki67 ≤ 14%	2.17 (1.70, 2.68)	0.450
	Ki67 > 14%	2.16 (1.71, 2.65)	
Age < 46	ER-positive	1.81 (1.49, 2.23)	0.078
	ER-negative	1.97 ± 0.62	
	PR-positive	1.80 (1.46, 2.20)	0.016
	PR-negative	1.99 ± 0.58	
	HER2-positive	1.92 (1.50, 2.39)	0.589
	HER2-negative	1.82 (1.50, 2.23)	
	Ki67 ≤ 14%	1.80 (1.49, 2.34)	0.978
	Ki67 > 14%	1.89 ± 0.46	

In the subgroups stratified by menopausal status, pathological type did not exhibit a statistically significant effect on LHR levels in either the premenopausal or postmenopausal groups (Table 6).

**Table 6:** Analysis results of LHR values for menopausal subgroups.

Clinical Features	Group	LHR values	P value
Premenopausal	ER-positive	2.01 (1.57, 2.47)	0.425
	ER-negative	2.03 (1.60, 2.57)	
	PR-positive	2.00 (1.55, 2.47)	0.055
	PR-negative	2.06 (1.62, 2.64)	
	HER2-positive	2.00 (1.53, 2.49)	0.491
	HER2-negative	2.02 (1.59, 2.48)	
	Ki67 ≤ 14%	2.04 (1.56, 2.51)	0.265
	Ki67 > 14%	1.94 (1.62, 2.38)	
Postmenopausal	ER-positive	2.26 (1.80, 2.71)	0.378
	ER-negative	2.31 ± 0.71	
	PR-positive	2.28 (1.81, 2.75)	0.651
	PR-negative	2.24 (1.77, 2.75)	
	HER2-positive	2.30 ± 0.66	0.566
	HER2-negative	2.26 (1.77, 2.76)	
	Ki67 ≤ 14%	2.26 (1.79, 2.75)	0.828
	Ki67 > 14%	2.29 ± 0.67	

In the subgroup of patients with a BMI ≥ 25 kg/m^2^, LHR levels did not differ significantly between the groups. Conversely, within the subgroup of patients with BMI < 25 kg/m^2^, PR-positive individuals exhibited significantly lower LHR levels than PR-negative patients, the median difference is −0.07. (95% CI: −0.12–-0.02) (Table 7).

**Table 7:** Analysis results of LHR values for BMI subgroups.

Clinical Features	Group	LHR values	P value
≥25 kg/m^2^	ER-positive	2.31 (1.90, 2.77)	0.142
	ER-negative	2.42 ± 0.75	
	PR-positive	2.31 (1.90, 2.76)	0.062
	PR-negative	2.43 ± 0.76	
	HER2-positive	2.39 ± 0.74	0.635
	HER2-negative	2.32 (1.90, 2.80)	
	Ki67 ≤ 14%	2.36 (1.89, 2.88)	0.147
	Ki67 > 14%	2.26 (1.90, 2.73)	
<25 kg/m²	ER-positive	1.99 (1.53, 2.45)	0.090
	ER-negative	2.08 ± 0.61	
	PR-positive	1.97 (1.53, 2.45)	0.045
	PR-negative	2.04 (1.64, 2.49)	
	HER2-positive	2.04 (1.59, 2.45)	0.372
	HER2-negative	1.98 (1.55, 2.48)	
	Ki67 ≤ 14%	1.99 (1.56, 2.47)	0.745
	Ki67 > 14%	2.01 (1.56, 2.48)	

### Logistic regression analysis

Using ER-positive status as the dependent variable, univariate logistic regression analyses were conducted incorporating variables such as age, menopausal status, BMI, and LHR. The results, presented in Table 8, indicated that both age and menopausal status were significantly associated with ER-positive status, whereas the other variables did not demonstrate statistically significant associations. Subsequently, these significant indicators were included in a forward stepwise multivariate logistic regression analysis, which identified postmenopausal status as an independent protective factor for ER positivity. Based on the multivariate logistic regression findings, a receiver operating characteristic (ROC) curve was constructed to assess the diagnostic performance of menopausal status in predicting ER-positive status. The x-axis of the ROC curve represents the false positive rate (1 – specificity), and the y-axis represents the true positive rate (sensitivity), as shown in Fig 1. The area under the curve (AUC) was 0.560 (95% confidence interval [CI], 0.534–0.585; P < 0.001).

**Table 8:** Logistic regression analysis of multiple factors associated with ER-positive status in breast cancer patients.

Variable	Univariate analysis			Multivariate analysis		
	OR	95%CI	P	OR	95%CI	P
**Age**						
<46						
≥46	0.711	0.580-0.871	**0.001**			
**Menopausal status**						
Premenopausal						
Postmenopausal	0.613	0.509-0.738	**<0.001**	0.57	0.431-0.754	**<0.001**
**BMI**						
<25 kg/m²						
≥25 kg/m²	0.951	0.793-1.141	0.590			
**LHR**	0.880	0.776-1.010	0.070			

**Fig 1:**
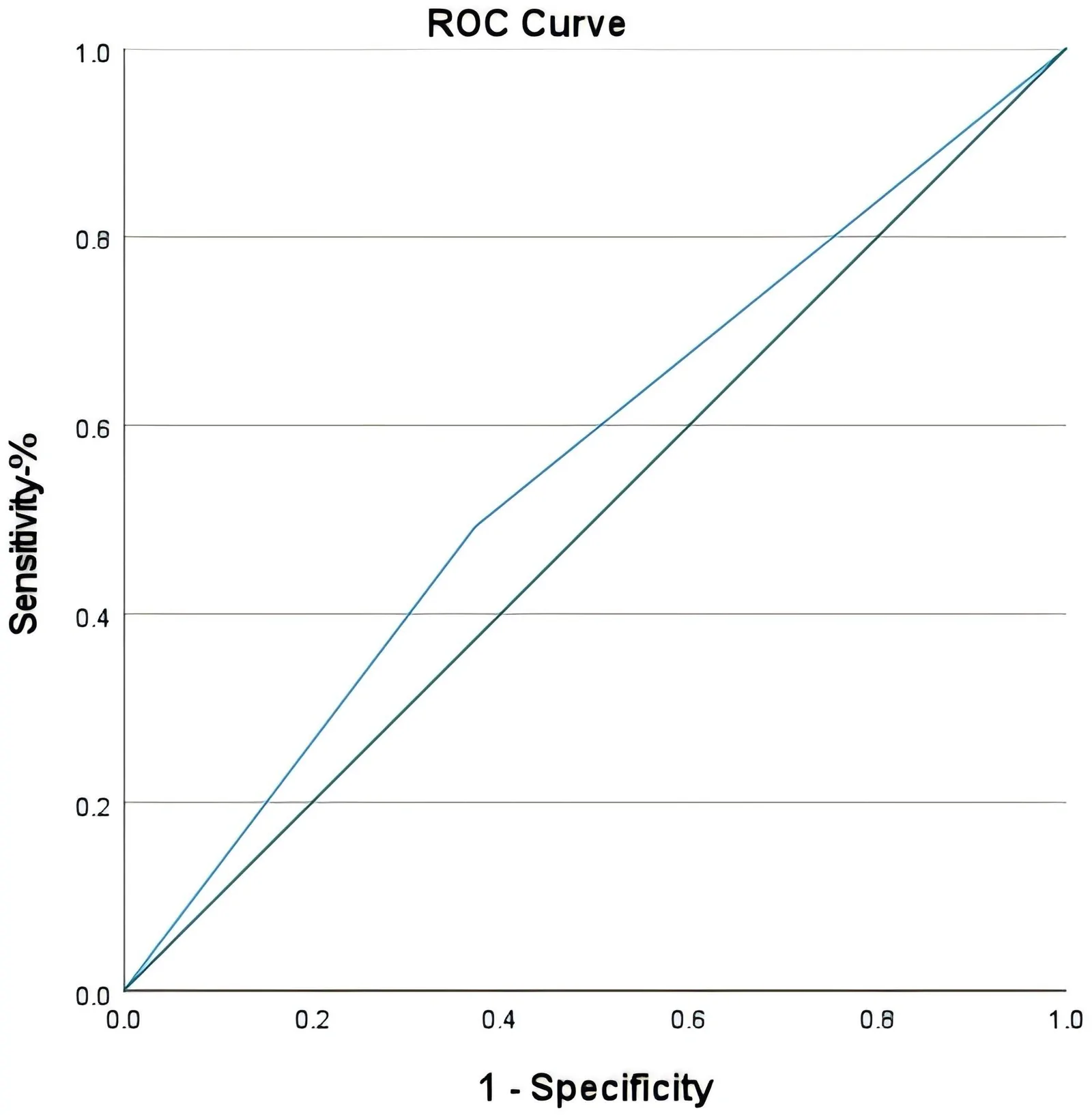
ROC curve of menopausal status for ER positive diagnosis.

Similarly, statistical analyses were performed on variables potentially influencing PR-positive status. The findings (Table 9) indicated that age, menopausal status, BMI, and LHR were significantly associated with PR-positive status. These variables were subsequently incorporated into a forward stepwise multivariate logistic regression analysis, which identified postmenopausal status as an independent protective factor for PR positivity. Based on the multivariate logistic regression results, a ROC curve was constructed (Fig 2) to assess the diagnostic performance of menopausal status in predicting PR-positive status, yielding an AUC of 0.592 (95% CI: 0.568–0.615, P < 0.001).

**Table 9:** Logistic regression analysis of multiple factors associated with PR-positive status in breast cancer patients.

Variable	Univariate analysis			Multivariate analysis		
	OR	95%CI	P	OR	95%CI	P
**Age**						
<46						
≥46	0.573	0.473-0.694	**<0.001**			
**Menopausal status**						
Premenopausal						
Postmenopausal	0.468	0.393-0.557	**<0.001**	0.492	0.370-0.654	**<0.001**
**BMI**						
<25 kg/m²						
≥25 kg/m²	1.634	1.380-1.935	**<0.001**			
**LHR**	0.860	0.761-0.972	**0.016**			

**Fig 2:**
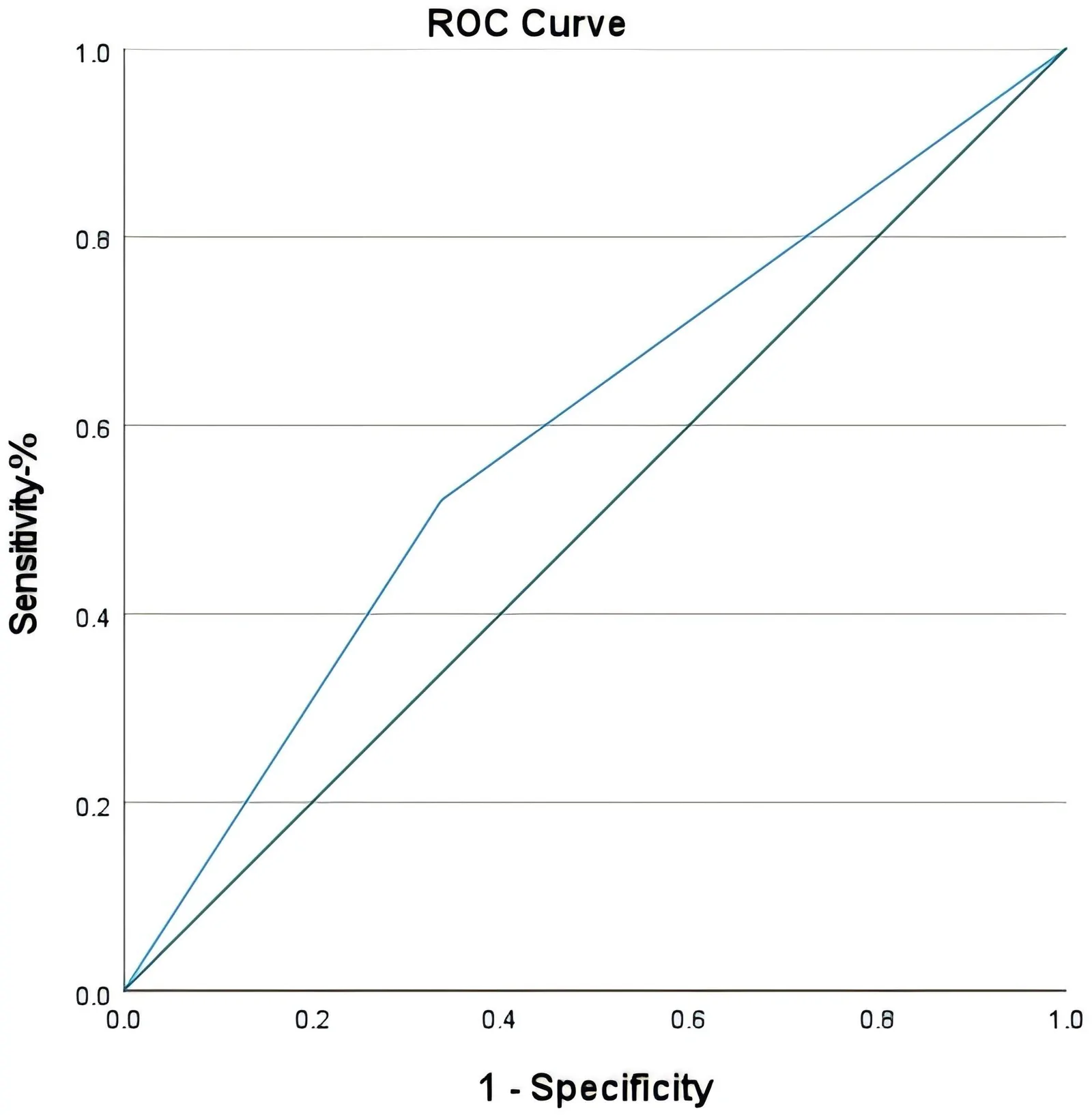
ROC curve of menopausal status for PR positive diagnosis.

A statistical analysis was performed to examine clinical characteristics and lipid profile indicators potentially associated with HER2-positive status. Univariate logistic regression analysis revealed that the clinical characteristics and LHR levels assessed did not significantly affect HER2-positive status in breast cancer patients (Table 10).

**Table 10:** Logistic regression analysis of multiple factors associated with HER2-positive status in breast cancer patients.

Variable	Univariate analysis		
	OR	95%CI	P
**Age**			
<46			
≥46	1.045	0.866-1.261	0.648
**Menopausal status**			
Premenopausal			
Postmenopausal	1.088	0.915-1.295	0.339
**BMI**			
<25 kg/m²			
≥25 kg/m²	0.858	0.720-1.022	0.086
**LHR**	1.006	0.886-1.142	0.927

## Discussion

This study systematically investigates the relationship between LHR and the clinicopathological characteristics of BC, with particular emphasis on its association with various molecular subtypes. The findings suggest that LHR is not solely indicative of systemic metabolic status but is also closely linked to the biological features of breast tumors, demonstrating subtype-specific patterns of association.

This study demonstrated that BC patients aged 46 years or older, those who are postmenopausal, and individuals with a BMI of 25 kg/m^2^ or higher exhibit elevated levels of the LHR, which is consistent with findings from prior research. Several biologically plausible mechanisms may underlie this association. Hhowever, these mechanisms remain speculative and cannot be conclusively determined based on our observational data. Aging is associated with a diminished hepatic capacity for lipid clearance and alterations in the activity of enzymes involved in lipoprotein metabolism, resulting in increased LDL-C and decreased HDL-C, thereby elevating LHR. The reduction in estrogen levels following menopause constitutes a principal factor that may drive the increase in LHR; estrogen upregulates hepatic LDL receptor expression and facilitates HDL synthesis, and its deficiency may directly contribute to the development of an atherogenic lipid profile. Furthermore, patients with a BMI ≥ 25 kg/m^2^ exhibit significantly elevated LHR, a phenomenon that may be linked to obesity-related insulin resistance, activation of inflammatory pathways, and adipose tissue dysfunction. Collectively, these factors may promote increased LDL-C and decreased HDL-C levels [29,30].

This study investigated the relationship between LHR levels and pathological characteristics of BC, revealing that patients with ER-positive and PR-positive BC exhibited significantly lower LHR levels compared to those with receptor-negative tumors, which is consistent with previous observations. Although the underlying mechanisms cannot be conclusively identified through this study, several plausible explanations have been suggested in the existing literature. First, hormone receptor-negative BC cells demonstrate rapid proliferation and high invasiveness, resulting in increased cholesterol demand, which may enhance LDL-C uptake through upregulation of LDL receptors [13,31,32]. Second, the LDL-C metabolite 27-hydroxycholesterol (27-HC) functions as an endogenous selective estrogen receptor modulator, with effects contingent upon ER status [33]. In ER-negative cells, 27-HC may exert its influence via non-estrogen receptor pathways [34]. Furthermore, compromised antioxidant and anti-inflammatory properties of HDL-C may contribute to the association between elevated LHR and more aggressive tumor phenotypes [35,36]. It is important to emphasize that these proposed mechanisms are intended to generate hypotheses and necessitate direct experimental validation. In this study, no significant difference in LHR levels was detected between patients exhibiting low versus high Ki67 expression, a finding that contrasts with some prior research. Ki67, as a marker of cellular proliferation, reflects tumor cell proliferative activity and is theoretically linked to lipid metabolic demands [37,38]. The absence of a significant difference in LHR levels may be attributable to the multifactorial regulation of Ki67 expression, which complicates the ability of lipid profile indicators alone to fully represent its variability. Additionally, the cutoff values employed to define high and low Ki67 expression in this study may have influenced the grouping outcomes. Patients with tumor size exceeding 2 cm demonstrated elevated LHR levels, suggesting that primary tumor growth may depend more heavily on cholesterol supply derived from LDL-C rather than on energy substrates from remnant lipoproteins. However, these differences were numerically small, with substantial overlap in distributions between groups, suggesting limited clinical meaningfulness despite statistical significance. Moreover, no significant association was observed between lymph node metastasis status and LHR levels, potentially indicating that the metastatic process is governed by more complex biological mechanisms that lipid metabolism indicators alone cannot adequately capture.

Age-stratified subgroup analysis revealed that the LHR was significantly elevated in PR-negative patients compared to PR-positive patients exclusively among individuals younger than 46 years. No such association was detected in patients aged 46 years or older. This observation implies that, in younger breast cancer patients, lipid metabolism may be more directly associated with hormone receptor status, whereas in older patients, age-related metabolic alterations may attenuate or obscure this relationship. Subgroup analysis based on menopausal status did not demonstrate a significant effect of pathological subtype on blood lipid-derived indicators, potentially due to the strong association between menopausal status and age. Consequently, age stratification may partially capture the influence of menopausal status. Furthermore, BMI-based subgroup analysis indicated that, among patients with a BMI ≥ 25 kg/m^2^, those exhibiting high Ki67 expression had significantly higher LHR levels than those with low expression. Conversely, in patients with a BMI < 25 kg/m^2^, PR-positive individuals exhibited significantly lower LHR levels compared to PR-negative individuals. These findings suggest that an obese metabolic background may modify the association patterns between blood lipid indicators and pathological characteristics. Specifically, in the high BMI cohort, blood lipid indicators appear to more closely reflect tumor proliferative activity, whereas in the normal-weight cohort, these indicators are more strongly related to hormone receptor status. These observations enhance the understanding of the heterogeneity in tumor biological behavior across various metabolic contexts. However, they are preliminary and necessitate validation in independent cohorts.

Although univariate analysis demonstrated an association between LHR and certain clinicopathological features, multivariate logistic regression analysis revealed that menopausal status, rather than LHR, was the sole independent predictor of ER-positive and PR-positive status. LHR did not achieve statistical significance in the multivariate models for ER, PR, or HER2 positivity. Accordingly, LHR cannot be regarded as a predictive factor for these outcomes, and thus, ROC analysis for LHR was not conducted. These findings suggest that the observed association between LHR and receptor status is likely confounded by age and menopausal status. Therefore, LHR should not be considered an independent predictive biomarker for receptor status. From a research perspective, several aspects of this study warrant careful consideration. First, LHR is a derived indicator that can be calculated from routine blood lipid tests without incurring additional costs, which facilitates its evaluation in large-scale epidemiological studies. Second, the observed associations, although modest, indicate that lipid metabolism may have a context-dependent role in BC biology, an area that is not yet fully understood. However, any potential clinical applications necessitate validation through studies that incorporate comprehensive control of confounding variables. Such investigations should emphasize the incremental value of LHR when integrated with established clinical and pathological factors, rather than evaluating its utility as an independent biomarker.

Despite these comprehensive findings, several limitations warrant consideration. First, the study’s single-center, retrospective design may restrict the generalizability of the results. Second, only a single preoperative lipid measurement was utilized. Dynamic monitoring of lipid fluctuations during treatment and postoperative periods, as well as their association with long-term prognosis, was not conducted. Third, although stringent exclusion criteria were employed to reduce confounding, residual confounding from unmeasured genetic, dietary, or lifestyle factors cannot be entirely excluded. Fourth, and importantly, the observational design of this study precludes any definitive conclusions regarding causality or the underlying biological mechanisms. Fifth, the near-chance AUC values indicate that LHR alone demonstrates insufficient discriminative performance for clinical application. Finally, the modest effect sizes observed in group comparisons suggest that the statistical significance identified in this study, which may be partially attributable to the relatively large sample size, does not necessarily imply clinical relevance. Future research should adopt a prospective, multicenter design with longitudinal follow-up to validate these findings. More comprehensive studies are warranted to further examine the dynamic changes in LHR throughout treatment and disease progression, as well as to assess the effects of lipid-lowering interventions on BC outcomes.

Based on the findings of the present study, future research will involve large-scale prospective cohort studies, commencing with baseline blood lipid measurements prior to BC diagnosis. The objective is to systematically assess the association between LHR and BC incidence, pathological characteristics, treatment response, and long-term prognosis. Additionally, a multidimensional risk assessment model integrating clinical and pathological features will be developed. Concurrently, the study will explore more profound associations between various blood lipid-derived indicators and BC molecular subtypes by employing multi-omics technologies to elucidate the underlying molecular mechanisms. Future research should investigate whether LHR offers additional predictive value when integrated with established clinical factors, rather than evaluating it solely as an independent biomarker.

## Supporting information

S1 File Original data. (XLSX)

S2 File Stepwise exclusion count. (XLSX)
